# *CpCAF1* from *Chimonanthus praecox* Promotes Flowering and Low-Temperature Tolerance When Expressed in *Arabidopsis thaliana*

**DOI:** 10.3390/ijms241612945

**Published:** 2023-08-18

**Authors:** Yimeng Lv, Mingfang Xie, Shiqing Zhou, Bixia Wen, Shunzhao Sui, Mingyang Li, Jing Ma

**Affiliations:** Chongqing Engineering Research Centre for Floriculture, Key Laboratory of Horticulture Science for Southern Mountainous Regions, Ministry of Education, College of Horticulture and Landscape Architecture, Southwest University, Chongqing 400716, China; iamiiim@163.com (Y.L.); xmf1709@163.com (M.X.); 393069965qqcom@sina.com (S.Z.); bx.wen@cas-envision.com (B.W.); sszcq@swu.edu.cn (S.S.); limy@swu.edu.cn (M.L.)

**Keywords:** *CpCAF1*, wintersweet, Arabidopsis, floral development, low-temperature tolerance

## Abstract

CCR4-associated factor I (CAF1) is a deadenylase that plays a critical role in the initial step of mRNA degradation in most eukaryotic cells, and in plant growth and development. Knowledge of *CAF1* proteins in woody plants remains limited. Wintersweet (*Chimonanthus praecox*) is a highly ornamental woody plant. In this study, *CpCAF1* was isolated from wintersweet. *CpCAF1* belongs to the DEDDh (Asp-Glu-Asp-Asp-His) subfamily of the DEDD (Asp-Glu-Asp-Asp) nuclease family. The amino acid sequence showed highest similarity to the homologous gene of *Arabidopsis thaliana*. In transgenic Arabidopsis overexpressing *CpCAF1*, the timing of bolting, formation of the first rosette, and other growth stages were earlier than those of the wild-type plants. Root, lateral branch, rosette leaf, and silique growth were positively correlated with *CpCAF1* expression. *FLOWERING LOCUS T (FT)* and *SUPPRESSOROF OVEREXPRESSION OF CO 1* (*SOC1*) gene expression was higher while *EARLY FLOWERING3 (ELF3)* and *FLOWERING LOCUS C* (*FLC*) gene expression of transgenic Arabidopsis was lower than the wild type grown for 4 weeks. Plant growth and flowering occurrences were earlier in transgenic Arabidopsis overexpressing *CpCAF1* than in the wild-type plants. The abundance of the *CpCAF1* transcript grew steadily, and significantly exceeded the initial level under 4 °C in wintersweet after initially decreasing. After low-temperature exposure, transgenic Arabidopsis had higher proline content and stronger superoxide dismutase activity than the wild type, and the malondialdehyde level in transgenic Arabidopsis was decreased significantly by 12 h and then increased in low temperature, whereas it was directly increased in the wild type. A higher potassium ion flux in the root was detected in transgenic plants than in the wild type with potassium deficiency. The *CpCAF1* promoter was a constitutive promoter that contained multiple *cis*-acting regulatory elements. The DRE, LTR, and MYB elements, which play important roles in response to low temperature, were identified in the *CpCAF1* promoter. These findings indicate that *CpCAF1* is involved in flowering and low-temperature tolerance in wintersweet, and provide a basis for future genetic and breeding research on wintersweet.

## 1. Introduction

The CCR4-associated factor 1 (CAF1) protein is a deadenylase and an essential component of the CCR4–NOT complex, which plays a fundamental role in eukaryotic mRNA metabolism and has a multitude of different roles that impact eukaryotic gene expression [1]. Plants can develop healthily and respond more quickly to environmental changes if their mRNA metabolism affects the expression level of particular genes. The majority of previous studies on the functional roles of *CAF1* are on yeasts and mammals [2,3,4,5]. The function of *CAF1* has been studied in certain plants, such as Arabidopsis, pepper (*Capsicum annuum*), and rice (*Oryza sativa*), which has revealed that the gene is vital for plant growth and development, as well as response to biotic and abiotic stresses [6,7,8,9]. The *Xanthomonas citri* (Xc) effector protein PthA4 decreases Cs*CAF1* deadenylase activity in Xc-infected leaves and stabilizes CsLOB1 mRNA in *Citrus sinensis* [10]. The authors reported that PthA4 increases *CsLOB1* transcription and translation to promote cell hypertrophy and hyperplasia in citrus by targeting the CCR4–NOT complex, implying that Cs*CAF1* limits cell growth in citrus. In Arabidopsis, hormones, cold damage, mechanical injury, and pathogen infection can cause the rapid expression of *CAF1* genes [11]. In rice, the regulatory elements in the *CAF1* promoter and variation in *CAF1* expression caused by hormones and nitrogen deficiency have been studied. Only *TaCAF1Ia*, a novel non-typical member of the *CAF1* subfamily that lacks the DEDD domain, may be a novel gene involved in another development. However, the suggested potential importance of typical *CAF1* proteins in plant reproductive development has not been confirmed and their precise mechanism is currently unknown [12].

Low temperature influences plant growth, development, yield [13], and geographical distribution [14]. Frost and extreme low temperature in winter limit the sustainable growth of trees. Freezing damage is a persistent impediment to tree growth in a cold-winter climate. Many species have developed mechanisms to tolerate low-temperature conditions. Blooming in winter with strong fragrance, *Chimonanthus praecox* (wintersweet) is a deciduous shrub with high ornamental and economic value [15]. The reliable effects of low temperature on flower bud differentiation and blossoming is important for the regulation of flowering in wintersweet [16]. When the cumulative chilling requirement (CR) reached 570 chill units (CU), chilling caused an upregulation of ABA levels and a significant downregulation of SHORT VEGETATIVE PHASE (SVP) and FLOWERING LOCUS T (FT) homologs at the transcript level in opened and unopened flower buds (FBs, anther and ovary differentiation completed), which suggested that dormancy breaking of FBs could be regulated by the ABA-mediated SVP-FT module [17]. The molecular mechanisms of cold tolerance and flowering of wintersweet are currently under investigation. For example, the ectopic overexpression of *CpNAC68*, a transgene from wintersweet, in Arabidopsis enhanced the tolerance of transgenic plants to cold environments, yet had no effect on growth and development [18]. And, the overexpression of *CpSIZ1*, a SIZ/PIAS-Type SUMO E3 ligase from wintersweet, could delay flowering time and enhance cold tolerance in Arabidopsis [18]. Previous research has demonstrated that under *CAF1* overexpression in Arabidopsis, rice, pepper, and other crops, the gene is actively expressed under low-temperature stress, effectively protecting the cell membranes and thus improving the cold tolerance of transgenic plants [19,20,21], although it has no effect on flower development. Therefore, it is probable that the biological traits caused by *CAF1* in wintersweet are unique.

The aim of the present study was to explore the functions of *CpCAF1* in wintersweet to gain insight into its regulatory mechanism. The full-length cDNA and upstream sequence of the *CpCAF1* gene were cloned. The tolerance of transgenic Arabidopsis and the physiological effects of *CpCAF1* expression in wintersweet were assessed. In addition, the upstream sequence promoter of *CpCAF1* was cloned and analyzed. The results revealed that *CpCAF1* plays an important role in low-temperature tolerance, plant growth, and flower development. The findings improve our understanding of how wintersweet is able to combat adverse environmental conditions and the mechanism of low-temperature-acclimation-induced low-temperature tolerance and flowering. Such knowledge is beneficial for breeding programs to enhance the ornamental value of wintersweet.

## 2. Results

### 2.1. Sequence Analysis of CpCAF1

The full-length cDNA of *CpCAF1* (accession no: OP714123) was 1106 bp and contained an 807 bp long ORF encoding a protein of 269 amino acid residues with a calculated molecular mass of 30.37 kDa. The *CpCAF1* protein contained a conserved domain (DEDD), including three aspartic acid (D), one glutamic acid (E), and several adjacent histidine residues belonging to the DEDDh subfamily of the DEDD RNase family.

**Figure 1:**
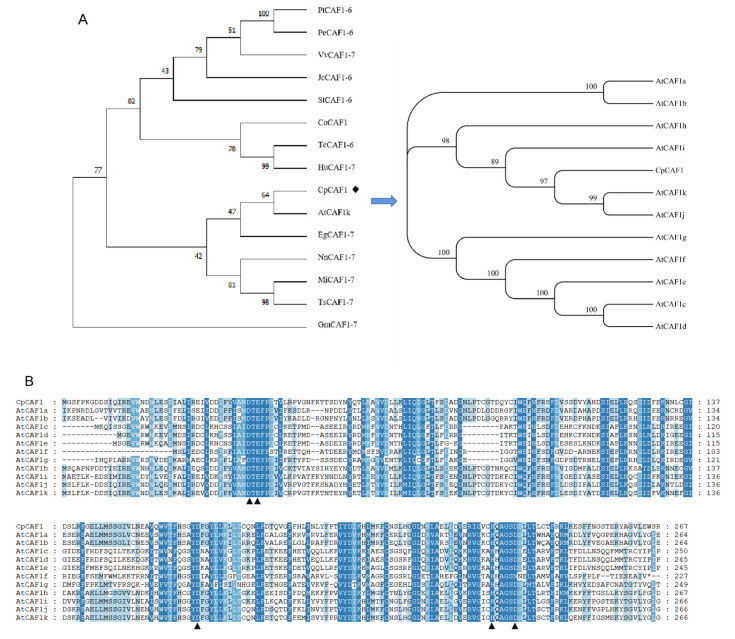
Multiple sequence alignment and phylogenetic relationships of wintersweet *CpCAF1*. (**A**) Neighbor-joining tree for plant *CAF1* proteins. The accession numbers from the GenBank of the sequences are as follows: *Populus trichocarpa* PtCAF1-6 (XP 024453230.1), *Populus euphratica* PeCAF1-6 (XP 011034532.1), *Vitis vinifera* VvCAF1-7 (XP 002272165.1), *Jatropha curcas* JcCAF1-6 (XP 012067515.1), *Senna tora* StCAF1-6 (KAF7814488.1), *Corchorus olitorius* Co*CAF1* (OMO74191.1), *Theobroma cacao* TsCAF1-7 (XP 007023403.2), *Herrania umbratica* HuCAF1-7 (XP 021298364.1), *Arabidopsis thaliana* AtCAF1k (NP 565735.1), *Elaeis guineensis* EgCAF1-7 (XP 010929136.1), *Nelumbo nucifera* NnCAF1-7 (XP 010275748.1), *Macadamia integrifolia* MiCAF1-7 (XP 042480429.1), *Telopea speciosissima* TsCAF1-7 (XP 043695888.1), and *Glycine max* GmCAF1-7 (XP 003522433.1). (**B**) Multiple alignment of the deduced amino acid sequence of *CpCAF1* and that of *CAF1* proteins from *Arabidopsis thaliana*. Blue boxes indicate identical amino acids. The DEDDh catalytic site residues are indicated with arrowheads. AtCAF1a (AT3G44260.1), AtCAF1b (AT5G22250.1), AtCAF1c (AT1G27820.1), AtCAF1d (AT1G27890.1), AtCAF1e (AT1G61470.1), AtCAF1f (AT3G44240.1), AtCAF1g (AT1G06450.1), AtCAF1h (AT1G15920.1), AtCAF1i (AT5G10960.1), AtCAF1j (AT1G80780.3).

**Figure 2:**
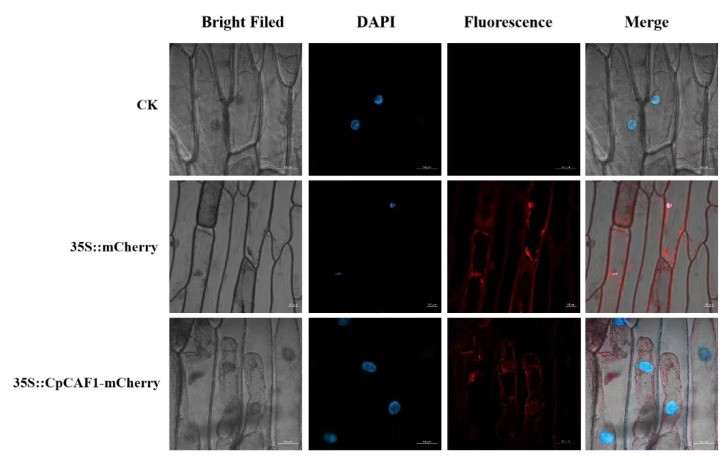
Subcellular localization of 35S::CpCAF1-mCherry in onion epidermal cells. Transient expression of 35S::mCherry and 35S::CpCAF1-mCherry fusion constructs in onion epidermal cells. Cells were analyzed with confocal microscopy 36 h after bombardment. Images were taken under bright light vision (**left**) and fluorescence field vision (**center**). Merged images are shown in the right-hand panel. The blue fluorescence shows DAPI as a nuclear localization assay and the red fluorescence shows the subcellular location of the target protein. Bar = 50 μm.

It is speculated that the gene encodes a relatively stable hydrophobic protein, which is an important feature of biofilm proteins. The results of a neighbor-joining analysis (Figure 1A) showed that the *CpCAF1* protein was highly similar to other *CAF1* proteins. For instance, the *CpCAF1* sequence showed 80–85% similarity with the *CAF1* proteins of *Arabidopsis thaliana*, *Nelumbo nucifera*, *Theobroma cacao*, *Vitis vinifera*, *Populus trichocarpa*, *Glycine max*, *Senna tora*, and *Corchorus olitorius*. It indicated that *CpCAF1* was more closely related to *Arabidopsis thaliana CAF1* than to the other homologous proteins. Further homologous protein amino acid sequence alignment of *CpCAF1* with 11 CAF1s of Arabidopsis marked a high degree of similarity (Figure 1B). The above conclusion was supported. No signal peptides were predicted in the sequence. The *CpCAF1* is most likely to be found in the nucleus, according to the results of subcellular localization prediction. Confocal microscopic analysis showed that *CpCAF1*-mCherry had the fluorescence signals in both the nucleus and cytoplasm (Figure 2).

### 2.2. CpCAF1 Response to Chilling and Flower Development in Wintersweet

**Figure 3:**
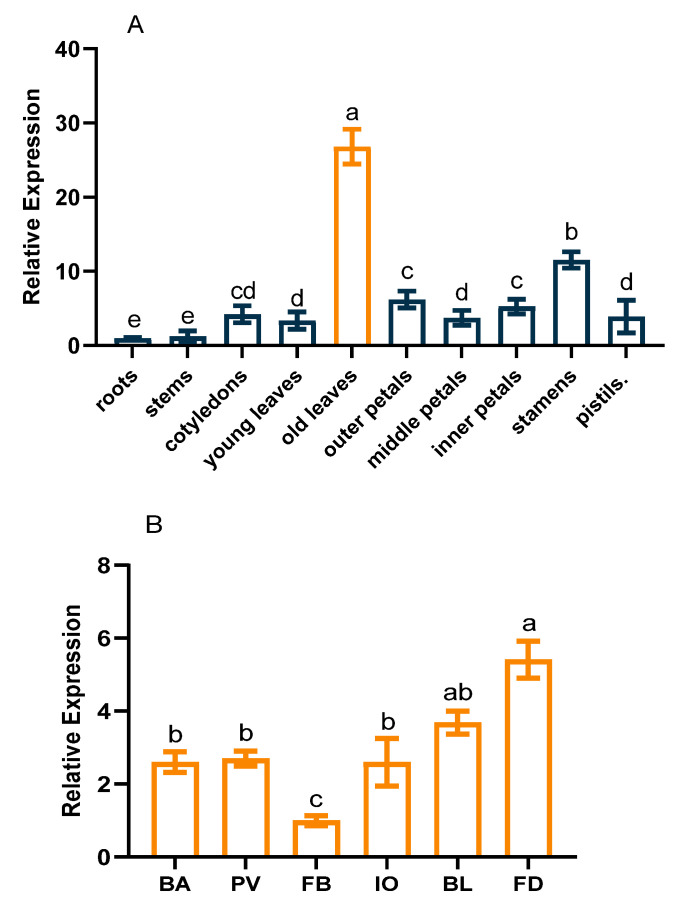
*CpCAF1* expression in different tissues and stages of floral development in wintersweet. (**A**) Relative expression of *CpCAF1* in different tissues. (**B**) Relative expression of *CpCAF1* at different stages of flower development (BA, flower buttress arises; PV, petals visible; FB, flower bud; IO, initial opening; BL, full bloom; FD, flower senescence). Each bar and error bar indicate the mean ± S.E. (*N* = 9). Results were analyzed using an analysis of variance, and a pairwise analysis of each event was performed using Duncan’s test for multiple comparison. The a–e represents the significance of differences analyzed applying Duncan’s multiple range test (α = 0.05).

A real-time quantitative PCR analysis revealed that *CpCAF1* was expressed in all analyzed tissues, comprising roots, stems, cotyledons, young leaves, old leaves (mature leaves), outer petals, middle petals, inner petals, stamens, and pistils. However, the relative expression levels showed tissue-specific variation (Figure 3A). The highest expression level was detected in old leaves, followed by stamens, and expression was lowest in roots. The expression level in old leaves was more than 26 times that in roots. Similarly, during flower development, the expression level was lowest in the flower bud stage and peaked at flower senescence (Figure 3B). The expression level in the flower senescence stage was more than 4 times that in the flower bud stage.

**Figure 4:**
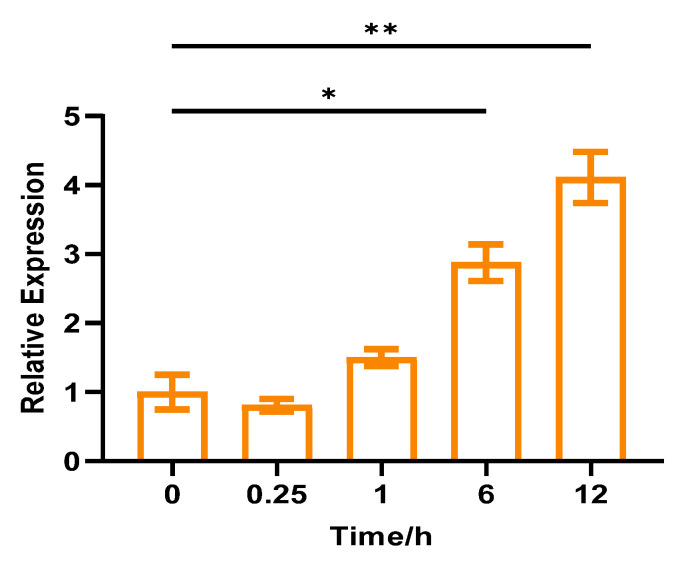
Relative expression of *CpCAF1* for low temperature (4 °C) in wintersweet. Each bar and error bar indicates the mean ± S.E. (*N* = 9). The significance of differences between means was assessed with one-way ANOVA using GraphPad Prism 9. * *p* < 0.05, ** *p* < 0.01.

The results suggested that *CpCAF1* transcription was strongly induced by 4 °C low-temperature exposure. Compared with the control, low-temperature treatment initially reduced the abundance of *CpCAF1* transcripts at 0.25 h, but thereafter the abundance gradually increased by 148% from 1 to 12 h and peaked at 12 h (Figure 4).

### 2.3. Overexpression of CpCAF1 in Arabidopsis

**Figure 5:**
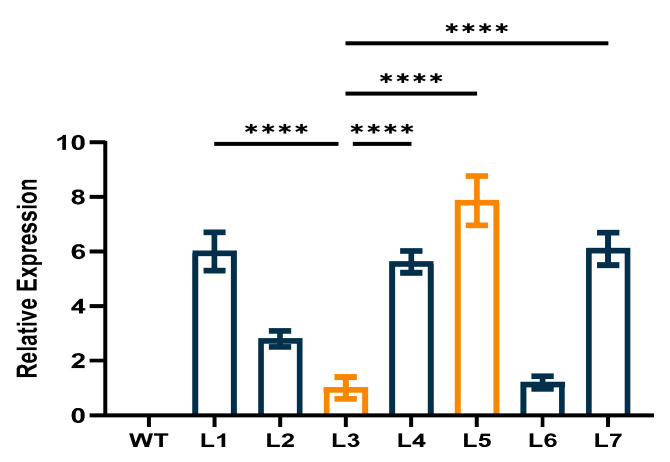
Relative expression of *CpCAF1* in leaves of transgenic *Arabidopsis thaliana* plants. WT, wild type (ecotype Col-0); L1–L7, *CpCAF1*-overexpressing lines. Each bar and error bar indicates the mean ± S.E. (*N* = 9). The significance of differences between L3 and the other group was assessed with one-way ANOVA using GraphPad Prism 9. **** *p* < 0.0001.

Seven homozygous transgenic Arabidopsis lines were screened in the T_4_ generation. Two overexpression lines (L5 and L3) were selected for further study because they showed the highest and lowest levels of *CpCAF1* expression, respectively, based on the qRT-PCR results (Figure 5).

**Figure 6:**
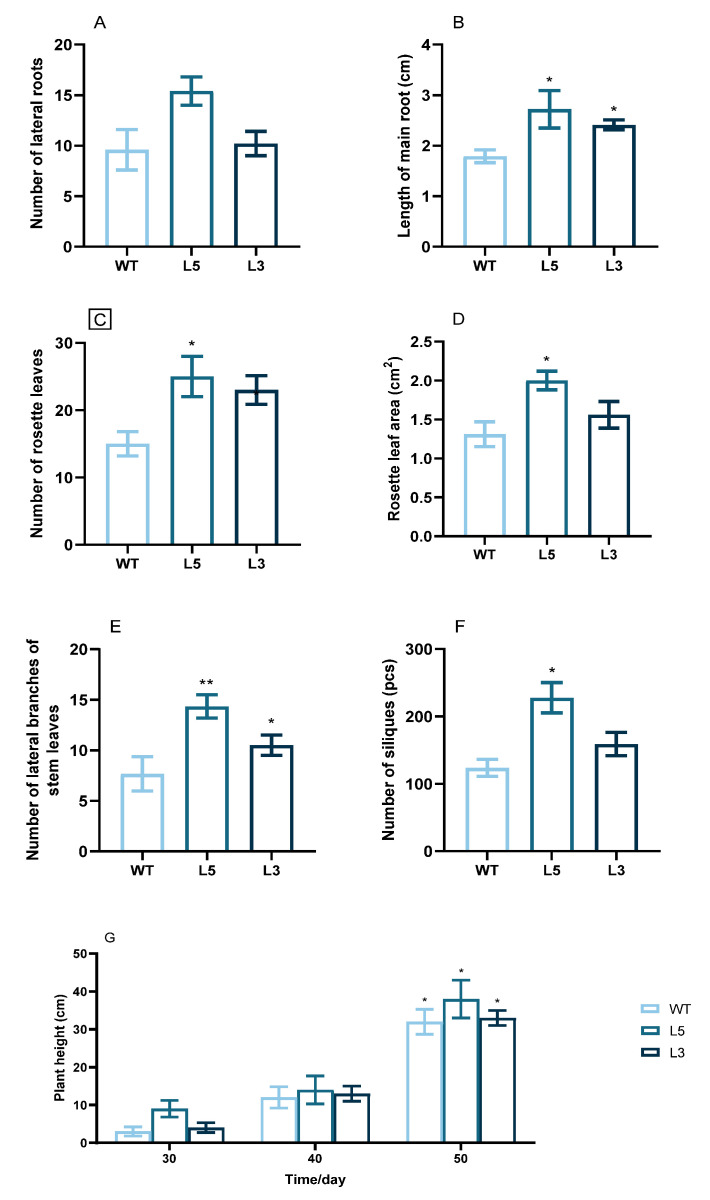
Phenotypic traits of two T_4_ transgenic lines of *Arabidopsis thaliana* overexpressing *CpCAF1*. (**A**) Main root length after growth for 25 days. (**B**) Number of lateral roots after growth for 25 days. (**C**) Number of rosette leaves at 30 days. (**D**) Leaf area of rosette leaves at 30 days. (**E**) Lateral branch number of stem leaves at 30 days. (**F**) Number of siliques at 60 days. (**G**) Average plant height at different stages of growth. WT, wild type (ecotype Col-0); L3 and L5, *CpCAF1*-overexpressing lines. The mean of each column was compared with the mean of the WT, and each bar and error bar indicates the mean ± S.E. (*N* = 9). The significance of differences between the treatment and the control group was assessed with one-way ANOVA using GraphPad Prism 9. * *p* < 0.05, ** *p* < 0.01.

Among the phenotypic traits of L5 and L3, the timing of bolting, formation of the first rosette and stem leaves, the first inflorescence, the first flower, and the first silique were earlier than those of the wild-type plants (Table 1). Root growth, lateral branch growth, rosette leaf growth, and silique growth of the transgenic lines were positively correlated with *CpCAF1* expression (Figure 6). It is noteworthy that, by the 30th day, the number of rosette leaves had reached 25 and 23 in L5 and L3, respectively, and flower opening had already occurred, whereas in the WT it had not (Figure 6C and Table 1).

**Figure 7:**
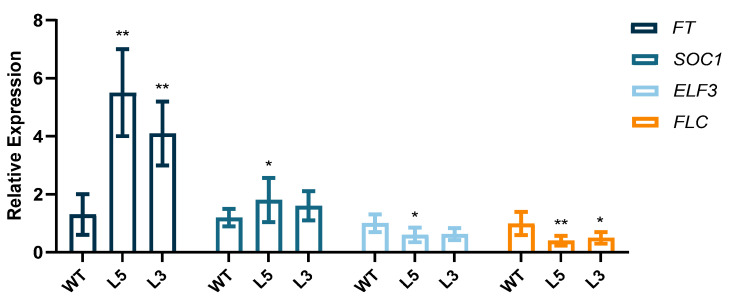
Relative expression levels of *FT*, *SOC1*, *ELF3*, and *FLC* in WT, wild type (ecotype Col-0) and L3 and L5, *CpCAF1*-overexpressing lines grown for 4 weeks. Compare the mean of each column with the mean of WT and each bar and error bar indicates the mean ± S.E. (*N* = 9). The significance of differences between the treatment and the control group was assessed with one-way ANOVA using GraphPad Prism 9. * *p* < 0.05, ** *p* < 0.01.

To investigate the effect of *CpCAF1* on flowering time in transgenic Arabidopsis, plants grown for four weeks were sampled to detect the expression of genes related to flowering regulation. Plants were grown under short day (SD) conditions for 4 weeks. The expression levels of *FT* and *SUPPRESSOROF OVEREXPRESSION OF CO 1* (*SOC1*) were increased significantly in L3 and L5, *CpCAF1*-overexpressing lines, whereas the expression levels of *EARLY FLOWERING3 (ELF3)* and *FLOWERING LOCUS C* (*FLC*) were decreased significantly in *CpCAF1*-overexpressing lines (Figure 7).

**Figure 8:**
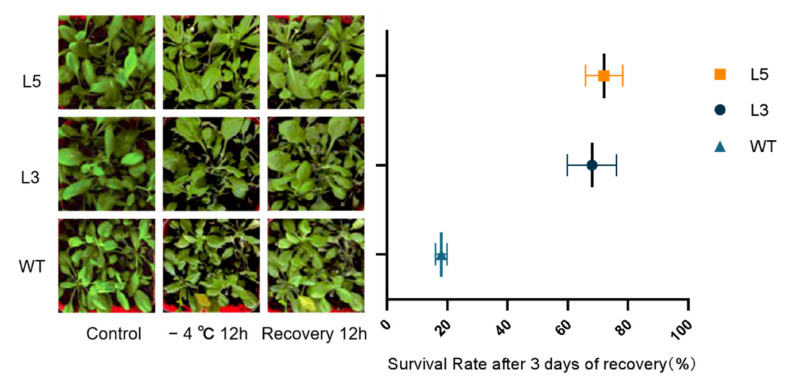
Phenotype and survival rate of two T_4_ transgenic lines of *Arabidopsis thaliana* overexpressing *CpCAF1* lines and wild type (WT) for low-temperature treatment and after recovery. Compare the mean of each plot with the mean of WT, and each bar and error bar indicates the mean ± S.E. (*N* = 9).

Different levels of low-temperature damage were inflicted on wild-type and transgenic Arabidopsis after 12 h of low-temperature (−4 °C) exposure and 12 h of recovery thereafter (Figure 8). Wilting and yellowing were observed, but the wilting in transgenic lines was less severe than in Arabidopsis of the wild type. After 3 days of recovery, the survival rates of L5 were 72.4% and 68.2%, which were significantly much higher than the WT survival rate of 18.9%.

### 2.4. Biochemical Analysis of Transgenic Arabidopsis Overexpressing CpCAF1

**Figure 9:**
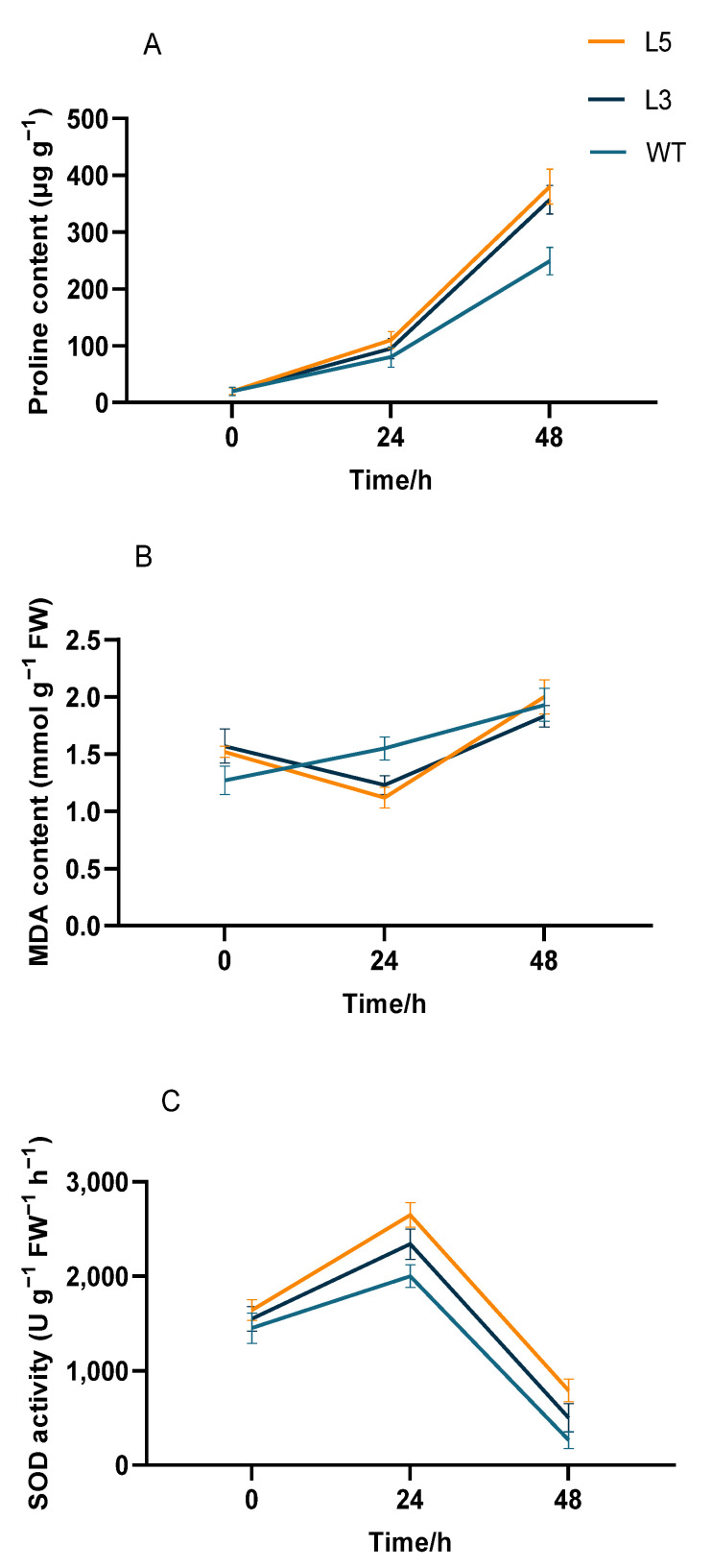
Physiological indicators in two T_4_ transgenic lines of *Arabidopsis thaliana* overexpressing *CpCAF1* under cold exposure. (**A**) Proline content, (**B**) malondialdehyde (MDA) content, and (**C**) superoxide dismutase (SOD) activity. WT, wild type (ecotype Col-0); L3 and L5, *CpCAF1*-overexpressing lines. Each bar and error bar indicates the mean ± S.E. (*N* = 9).

Notable differences in proline content, MDA content, and SOD activity were observed between untreated transgenic Arabidopsis overexpressing *CpCAF1* and wild-type plants for low-temperature treatment. The proline content of all plants gradually increased in response to 4 °C treatment, but that of transgenic Arabidopsis was significantly higher than that of the wild type (Figure 9A). At 48 h, the proline content of the strongly *CpCAF1* overexpressing L5 line increased by 19 times, whereas that of the weakly *CpCAF1* overexpressing L3 line increased by 17 times and the proline content of the wild type increased by 12.45 times. The proline content of L5 plants was 53.80% higher than the wild type and that of the L3 plants was 44.19% higher than the wild-type plants. The wild type suffered irreversible damage after 4 °C treatment as the MDA content increased. In the L5 and L3 lines, the MDA content decreased by 26.3% and 21.6% at 24 h and then increased at 48 h, and the plants suffered irreversible damage from low-temperature treatment (Figure 9B).

**Figure 10:**
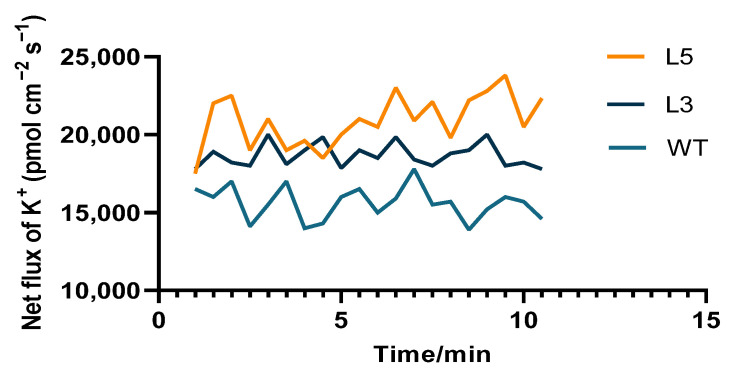
Net flux of K^+^ in root tips of two T_4_ transgenic lines of *Arabidopsis thaliana* overexpressing *CpCAF1* and the wild type. WT, wild type (ecotype Col-0); L3 and L5, *CpCAF1*-overexpressing lines (*N* = 9).

The SOD activity of all plants was increased in response to low-temperature treatment (Figure 9C). After 24 h of low-temperature treatment, the SOD activity of the L5 line was the highest and had increased by 67.0%. The SOD activity of the L3 line had increased by 51% and that of the wild type increased by 37.9%. The SOD activity of the L5 line was 32.5% higher than the wild type and that of the L3 was 17.0% higher than the wild type. After low-temperature treatment for 48 h, all plants showed decreased SOD activity, but the L5 and L3 transgenic Arabidopsis plants retained higher activity than the wild type. The potassium influx of the transgenic lines and wild type was induced to varying degrees with potassium deficiency. The potassium influx in mature root tissues of strongly *CpCAF1* overexpressing L5 seedlings attained approximately 21 pmol cm^−2^ s^−1^, whereas that of weakly *CpCAF1* overexpressing L3 seedlings was approximately 18 pmol cm^−2^ s^−1^, which were both higher than that of wild-type seedlings (Figure 10).

### 2.5. Cloning, Sequence Analysis, and Functional Characterization of the Promoter of CpCAF1

The 2133 bp of the 5′-upstream promoter region of *CpCAF1* was cloned and designated *CpCAF1pro*. Sequence analysis showed that the TSS was located −225 bp upstream of the ATG and a map of the *cis*-acting regulatory elements in the promoter was constructed (Table 2). The AT1-motif, W-box, AuxRR-core, ABRE, SARE, TCA-motif, and other motifs play crucial roles in the responses to light, defense, auxin, abscisic acid (ABA), and salicylic acid. In addition, DRE (at position −456), LTR (at position −249), and MYB (at positions +151, −186, and −1231) domains, which are involved in the response to low-temperatures stress, were predicted.

**Figure 11:**
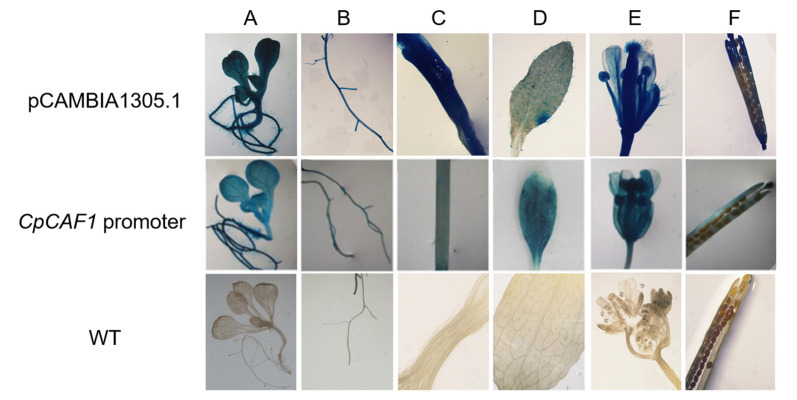
Histochemical analysis of GUS activity driven by the *CpCAF1* promoter in *Arabidopsis thaliana*. (**A**) Young seedling; (**B**) root; (**C**) stem; (**D**) leaf; (**E**) flower; (**F**) silique; *CpCAF1* promoter, GUS activity driven by the *CpCAF1* promoter in transgenic Arabidopsis; pCAMBIA1305.1, GUS activity driven by 35S of pCAMBIA1305.1 in transgenic Arabidopsis; WT, wild type (ecotype Col-0).

**Figure 12:**
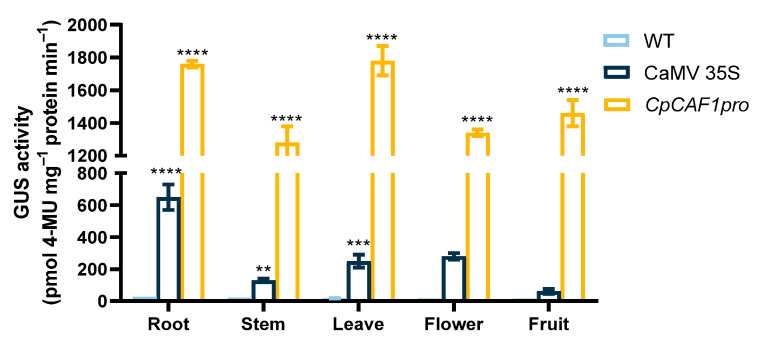
GUS activity driven by the *CpCAF1* or CaMV *35S* promoters in *Arabidopsis thaliana* organs. Each bar and error bar indicates the mean ± S.E. (*N* = 9). The significance of differences between the *CpCAF1* or CaMV *35S* promoters and the wild type was assessed with two-way ANOVA using GraphPad Prism 9. ** *p* < 0.01, *** *p* < 0.001, **** *p* < 0.0001.

To further explore the function of the *CpCAF1* promoter, the transgenic plants were screened for secondary culture. Histochemical analyses of T_2_ transgenic Arabidopsis lines during the full developmental period indicated that the *GUS* gene driven by the *CpCAF1* promoter was expressed in vegetative and reproductive organs (Figure 11). Quantitative fluorometric assays (Figure 12) were consistent with the results of the histochemical analysis, confirming that significant GUS activity was present in each of the major tissues and that the *CpCAF1* promoter was a strongly constitutive promoter.

**Figure 13:**
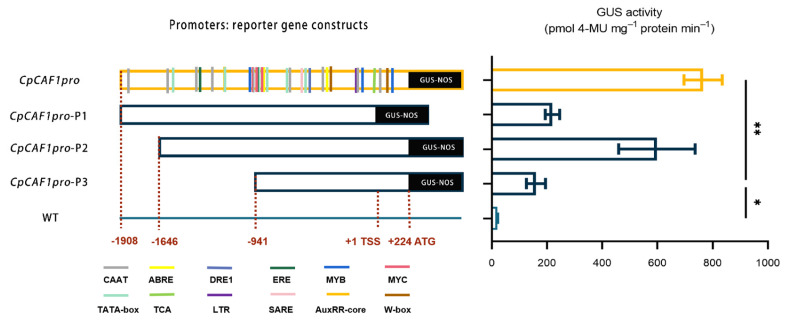
Schematic representation of the *CpCAF1* promoter and deletion fragment: reporter gene constructs and GUS activity in transgenic *Arabidopsis thaliana*. Each bar and error bar indicates the mean ± S.E. (*N* = 9). The significance of differences between the *CpCAF1pro*-P3 and the other group was assessed with one-way ANOVA using GraphPad Prism 9. * *p* < 0.05, ** *p* < 0.01.

The results of serial deletion analysis in transgenic Arabidopsis (Figure 13) indicated that the promoter fragments *CpCAF1pro* and *CpCAF1pro*-P1/P2/P3 drove significantly higher GUS activity than the wild type. The *CpCAF1pro* and *CpCAF1pro*-P2 promoter fragment-driven GUS activity was maintained at high levels and both were significantly higher than that of the *CpCAF1pro*-P3 fragment. The *CpCAF1pro*-P1 promoter fragment drove significantly lower GUS activity than the *CpCAF1pro* promoter fragment.

## 3. Discussion

The CCR4–NOT complex in yeast and humans is composed of nine protein subunits and functions as a deadenylase, with CCR4 playing a prominent role and CAF1 filling in the gaps. A previous study discovered a novel pattern of interaction between OsCCR4 and OsCAF1 in the rice CCR4–NOT complex, and that OsCAF1 acts as a bridge between OsCCR4 and OsNOT1 in this complex [22]. CAF1 is involved in the deadenylation of mRNA in plants [23], and regulates gene expression and influences biological features [24]. The CAF1 gene is also essential for yeast and animal cells to grow and develop appropriately, although they harbor only one or two CAF1 homologs, in contrast to higher plants, which have the whole CAF1 gene family [25]. A total of 18 CAF1 homologs have been identified in rice [26], whereas 11 CAF1 orthologs have been identified in Arabidopsis. The presence of the CAF1 gene sequence in Vitis vinifera, Sorghum bicolor, Oryza sativa, and Arabidopsis was confirmed via a full-sequence genomic BLAST search of GenBank for certain plants [27]. Several CAF1 proteins are vital for plant growth and play essential roles in stress response [2,28,29,30].The potential function of CAF1 in flower development, however, has been reported previously only once: TaCAF1Ia1, a novel non-typical CAF1 lacking the DEDD domain, is specifically expressed during the early stages of anther development in wheat (Triticum aestivum). It is assumed that a distinct miR2275–CAF1 pathway evolved to regulate reproductive development in wheat [31]. However, the precise mechanism is currently not fully understood. The role of typical CAF1 proteins in reproductive development requires further investigation.

In the present study, CpCAF1, which was isolated from a wintersweet cDNA library, contained one ORF encoding a protein that shared high homology with the CAF1 of many eukaryotic organisms, especially Arabidopsis (Figure 1). Because the functional roles of CAF1 in wintersweet have not been studied previously, we explored the roles of CpCAF1 using wintersweet and the overexpression of CpCAF1 in transgenic Arabidopsis. The CpCAF1 promoter was successfully cloned and analyzed. The cis-acting regulatory elements of the CpCAF1 promoter were preliminarily investigated.

A qRT-PCR assay was used to examine the expression patterns of *CpCAF1* in various organs and developmental stages of wintersweet (Figure 3A). The *CpCAF1* gene was expressed in all analyzed tissues. However, the expression levels differed during flower development (Figure 3B). Between the flower senescence stage and the flower bud stage, the expression level of *CpCAF1* showed an upward trend. It can be speculated that there may be unique traits and different mechanisms by which *CpCAF1* was involved in floral organ development or delayed flower growth. This study provides a crucial foundation for investigating the important and uncommon potential roles of *CAF1* in flower development. The present results also indicated that 4 °C treatment induced *CpCAF1* transcription. The optimum temperature for flowering in wintersweet is a cooler temperature of 4 to 10 °C; 4 °C treatment initially decreased *CpCAF1* transcript abundance, but subsequently the *CpCAF1* transcript level climbed steadily and exceeded the original level (Figure 4). The expression levels of *C-REPEAT BINDING FACTOR* (*CBF*), whose ectopic expression can compensate for the absence of cold acclimation, and *COLD-REGULATED* (*COR*) genes in Arabidopsis tended to stabilize at 20 °C before cold treatment, while gene expression showed a fold change increase when treated at 4 °C within a day. The increased expression of these genes during cold acclimation increases freezing tolerance [32,33]. Therefore, based on the results of this study, we can speculate that *CpCAF1* is implicated in chilling-response signaling pathways, which may be similar to many *CAF1* genes induced by low-temperature treatment, such as *AtCAF1* [34], *DsCAF1* [35], *OsCAF1B* [36], and Pt*CAF1* [28].

In the T_4_ transgenic lines of Arabidopsis overexpressing *CpCAF1*, important growth stages such as the timing of bolting, first rosette leaf branch, and first flower appeared earlier than that of wild-type plants. Furthermore, the expression of the transgenic Arabidopsis *FT* and *SOC1* gene was significantly much higher than that of the WT, whereas the expression of the *ELF3* and *FLC* gene was significantly lower than that of the WT (Figure 7). The FT, expressed in leaves, functions as a florigen that is transported to the shoot apex to induce flowering. SOC1 was shown to directly bind to the *FT* promoter to activate FT transcription, thus forming a positive feedback, feed-forward loop at the leaf and growing point FT-SOC1 to ensure plant flowering induction [37]. *FLC* is a key gene that suppresses flowering in plants. It regulates flowering by repressing the flowering regulatory elements *FT* and *SOC1* [38]. ELF3 is a negative regulator of flowering in Arabidopsis [39]. It indicates that *CpCAF1* accelerated the transgenic Arabidopsis growth cycle, and notably also promoted earlier flowering in reproductive growth, compared with the wild type. It supports the results that suggest that *GAF1* promotes the expression of *FT* and *SOC1* by repressing genes encoding negative regulators of flowering including *ELF3*, a component of the Evening Complex (EC) in GA-controlled flowering [40]. Additionally, it is speculated that *CAF1* may participate in the Arabidopsis flowering autonomous pathway by acting as a deadenylase, degrading the target gene’s (*FLC*) mRNA, lowering its abundance, reducing its negative regulatory impact on *FT* and *SOC1*, and encouraging flowering and the emergence of an early flowering phenotype. Another way *CAF1* may function similarly to FY in Arabidopsis is by regulating the alternative polyadenylation (APA) of *FLOWERING CONTROL LOCUS A (FCA)* and *FLC*, two genes involved in blooming. Based on root length and other phenotypic indicators (Table 1, Figure 6), we concluded that transgenic Arabidopsis traits outperform wild-type Arabidopsis traits and the traits were proportional to gene expression level. Consistent with the aforementioned findings, *CAF1* may play an important role in promoting plant growth.

To explore the changes in *CpCAF1* expression in a low-temperature environment, we comparatively analyzed the lines that showed the highest and lowest levels of *CpCAF1* expression (L5 and L3, respectively) for comparison in accordance with our previous research. Transgenic Arabidopsis showed strong low-temperature tolerance in the phenotype in a low-temperature environment (Figure 8). Proline accumulates in large amounts in plant tissues under certain stresses, protecting the cells against damage from reactive oxygen species [41], and is essential for osmoregulation [34,42]. The proline content of the high-*CpCAF1*-overexpressing L5 line showed the fastest and highest growth response to low-temperature treatment and improved the tolerance of cells to low-temperature stress (Figure 9A). Malondialdehyde is a cytotoxic product of lipid peroxidation that serves as a marker for free radical generation and tissue damage [43,44]. Under low-temperature stress, MDA, the product of membrane lipid peroxidation in plants, will accumulate in large quantities. Accumulation of MDA may cause some damage to the membrane and cells. With an increase in the duration of low-temperature treatment, the scavenging ability of the plant itself decreased and the MDA content increased. A slightly upward trend was observed for the MDA content in wild-type plants; however, the MDA content of transgenic Arabidopsis decreased initially and then increased, and the decrease in the high-*CpCAF1*-overexpressing L5 line was more obvious (Figure 9B). These results showed that the order of low-temperature tolerance of these three lines was L5 > L3 > wild type.

Superoxide dismutase is a metalloenzyme that catalyzes the dismutation of superoxide anions to oxygen and hydrogen peroxide. These responses serve as a defense strategy for the survival of an aerobic species [45]. The SOD activity in Arabidopsis leaves was increased by short-duration low-temperature treatment. The SOD activity of the L5 line increased the fastest and to the greatest extent, followed by the L3 line, and the difference in activity of the wild type was the smallest (Figure 9C). As low-temperature damage continues, SOD activity decreases due to the accumulation of superoxide anion free radicals and the broken balance of superoxide (O_2_^·−^) in the plant. In transgenic rice and pepper overexpressing CAF1, the gene is actively expressed in cold environments and the contents of proline and MDA, and SOD activity shows corresponding changes, thus effectively protecting the cell membranes and improving the cold tolerance of the transgenic plants [46]. The present results indicated that transgenic Arabidopsis plants were less damaged than wild-type or control plants, and thus they had superior cold tolerance, which was similar to the previous findings in rice and pepper. In wintersweet, CpNAC68 has a positive regulatory role in stress response processes. Under the stresses of heat, osmotic, and salt, the ectopic overexpression of CpNAC68 could decrease the relative electrolyte leakage and MDA content raised by transgenic Arabidopsis. The survival rate and chlorophyll content (SPAD values) were significantly greater than those of the WT plants [18,47]. According to our results, it is reasonable to speculate that *CAF1* genes are involved in plant cold tolerance regulatory pathways by increasing the proline content, enhancing SOD activity, and reducing the MDA content.

Potassium is a macroelement essential for plant growth and development. A crucial role for sufficient K^+^ nutrition in plant tolerance to various abiotic and biotic stresses has been reported [48]. According to the currently available evidence, potassium supports plant survival in cold environments by decreasing reactive oxygen species production and increasing antioxidant concentrations [36,49]. We used non-destructive microscopy to evaluate the potassium ion flow rate of Arabidopsis roots for approximately 2 weeks to explore the mechanism of action of *CpCAF1* on potassium ion absorption in Arabidopsis roots. In a low-potassium environment, the potassium influx of transgenic and wild-type seedlings was induced to varying degrees (Figure 10). The strongly *CpCAF1* overexpressing L5 line showed the greatest induction, followed by the weakly *CpCAF1* overexpressing L3 line, and induction was weakest in the wild type. Compared with the cold-sensitive tomato cultivar ‘S708′, the cold-tolerant tomato cultivar ‘T722’ exhibits a lower reduction in plant growth rate, whole-plant K^+^ content, and K^+^ net uptake [50]. Moreover, potassium functions in the plant signaling system to defend against low-temperature conditions and ensure an adequate potassium supply for the plant, whereas potassium deficiency can increase the susceptibility of plants to low temperatures [51]. The strongly *CpCAF1* overexpressing L5 transgenic Arabidopsis line exhibited a significant potassium-deficiency-induced potassium influx and greater potassium ion flow into the cell even with potassium deficiency. Thus, it is reasonable to speculate that *CpCAF1*-overexpressing Arabidopsis has a better potassium deficiency tolerance and superior growth for low-temperature exposure than the wild type. This suggests that *CpCAF1* is involved in the regulation of the potassium pathway to improve the low-temperature tolerance of plants.

In response to diverse environmental stimuli, plants have complex signal transduction networks and synthesize signal molecules for the defense response [46]. The *CpCAF1* promoter sequence contained DRE, LTR, and MYB elements, which are involved in the low-temperature stress response, ABRE elements induced by drought, MYC elements that play a role in drought stress response, and ABA induction (Table 2). The promoter regions of the 19 Pt*CAF1* genes of Populus trichocarpa contain many cis-acting elements responsive to environmental stress [52]. The plant hormone ABA plays important roles in plant development and response to stresses [53,54,55]. The present results suggest that *CpCAF1* plays a role in plant defense responses. A histochemical analysis of the construct containing the regulatory region of the *CpCAF1* promoter revealed GUS expression in all tissues examined (Figure 10 and Figure 11). However, the GUS activity driven by the *CpCAF1*pro-P1 fragment was much lower than that driven by *CpCAF1pro*. The *CpCAF1pro* and *CpCAF1pro*-P2 promoter fragment-driven GUS activity was maintained at high levels and was significantly higher than that of the *CpCAF1pro*-P3 fragment (Figure 13). These results indicated that putative region-specific enhancers may exist within the promoter from −1621 to −941 bp and 0 to +224 bp. The high AT content (70.9%) in the *CpCAF1* promoter sequence, which is generally accepted to be a characteristic of duplex DNA more prone to unwinding and improves the transcriptional efficiency of the gene [29], likely explains why the *CpCAF1* promoter promotes GUS activity more significantly than the 35S promoter.

## 4. Materials and Methods

### 4.1. Plant Materials, Growth Conditions, and Low-Temperature Treatment

Adult individuals of wintersweet (*C. praecox*) were planted in a nursery at Southwest University (Chongqing, China). *Arabidopsis thaliana* ecotype Columbia (Col-0) was used for genetic transformation. All Arabidopsis plants were grown at 25 °C under a 16 h/8 h (light/dark) photoperiod and a light intensity of 55 µmol·m^−2^·s^−1^. Murashige and Skoog (MS) medium [56] supplemented with kanamycin (100 mg L^−1^) was used to screen for transformants. Wintersweet seeds were germinated in pots containing nutritional soil and seedlings at the four-leaf stage were used in the following treatments. For the low-temperature treatment, the seedlings were placed in a growth cabinet maintained at 4 °C for 0.25, 1, 6, or 12 h; refer to the research results of Li et al. [17]. The cotyledons, young leaves, roots, stem, and leaves were collected separately from plants at the four-leaf stage for RNA isolation. Flower buds and whole flowers were collected at different stages of floral development from the adult plants, following the method of Sui et al. [57] for RNA extraction and gene expression analysis.

### 4.2. Cloning of CpCAF1 and Bioinformatic Analysis

A potential target expressed sequence tag (EST) sequence was isolated from the wintersweet flower EST library using the 5-pTriplEx2 sequence primer (5′-TCCGAGATCTGGACGAGC-3′) [58]. The BlastN (nucleotide–nucleotide BLAST) and BlastX (translated query vs. protein database) tools accessible on the NCBI website (http://www.ncbi.nlm.nih.gov/ (accessed on 15 January 2020) and SeqMan version 11.1 (DNASTAR, Inc., Madison, WI, USA) were applied to analyze multiple sequence alignments and cluster analysis on the EST sequence. The full-length cDNA of *CpCAF1* was cloned from the library with the primer pair T7 (5′-TAATACGACTCACTATAGGG-3′) and SP5 (5′-CTTCTGCTCTAAAAGCTGCG-3′). The sequence characteristics were analyzed with DNASTAR version 7.1 and DNAMAN version 6.03.99 bioinformatic software. The signal peptide and subcellular localization were predicted with the online software SignalP version 5.0 (http://www.cbs.dtu.dk/services/SignalP/ (accessed on 30 January 2020) and the TargetP 1.1 server (http://www.cbs.dtu.dk/services/TargetP/ (accessed on 30 January 2020), respectively. The alignment of the amino acid sequences of homologous proteins and the construction of a dendrogram using the neighbor-joining method were conducted with MEGA version 11 [59].

### 4.3. Subcellular Localization of CpCAF1

The coding sequence (CDS) of *CpCAF1* without stop codon was inserted into SacI and BamHI of the modified pCAMBIA1300-mCherry vector to form a recombinant vector *35S::CpCAF1-mCherry*. The epidermis of onion cells was cultured in MS media for 24 h in the dark, then agrobacterium GV3101 carrying 35S::*CpCAF1*-mCherry or 35S::mCherry were transferred into onion epidermal cells. After 36 h, mCherry fluorescent was observed using confocal laser microscopy (Olympus, Japan).

### 4.4. RNA Isolation and Quantitative Real-Time PCR Analysis of CpCAF1

Total RNA from all plant samples was isolated using the RNAprep Pure Kit (TIANGEN, Beijing, China). RNA sample OD260/OD280 and OD260/OD230 were determined with NanoDrop2000 (Thermo, Shanghai, China) and RNA quality was determined via agarose gel electrophoresis. The remnant genomic DNA from RNA was removed and the first-strand cDNA was synthesized using the All-in-One First-Strand Synthesis MasterMix (with dsDNase) (Yugong Biolabs, Jiangsu, China). The amount of RNA per PCR sample was 1 μg. Quantitative real-time PCR (qRT-PCR) was performed with the SsoFast™ EvaGreen SuperMix Kit (BioRad, Hercules, CA, USA). The primers used for real-time PCR were designed with Primer Premier 5.0. *CpActin* and were used as the internal reference genes. The reactions took place at a 20 μL volume and contained 4.0 μL 10 × each of forward and reverse primers, 2.0 μL 10× of cDNA, and 10.0 μL SsoFast™ EvaGreen^®^ Supermix. The reactions were carried out using the following protocol: 95 °C for 30 s, followed by 40 cycles of 95 °C for 5 s and 56 °C for 30 s, 65 °C for 0.05 s, and 95 °C for 0.5 s. qRT-PCR equipment was c1000 touch thermal cycler combined with CFC96^TM^ Real-Time system (BioRad, CA, USA). Relative gene expression levels were calculated using the 2^−ΔΔ*C*t^ method [60] with Bio-Rad Manager™ (Version 1.1) software. In addition, qRT-PCR was used to detect the *CpCAF1* expression level in transgenic Arabidopsis, relative to the *AtActin* reference gene, using the above-mentioned method. All primers used in the study are listed in Table S1. Three biological replicates and three technical replicates were analyzed for each qRT-PCR reaction.

### 4.5. Construction of Plant Overexpression Vectors for CpCAF1 and Transformation of Arabidopsis

The open reading frame (ORF) of *CpCAF1* was amplified with gene-specific primers (*CpCAF1*-F: 5′-GAATTCATGGGGAGTTTTCCCAAAGGTG-3′, *CpCAF1*-R: 5′-GGATCCTTACCACCGAGACCATTCCAAGACAC-3′) containing the restriction enzyme sites *Sac*I and *Xbal*I. The amplified fragments were inserted into the binary vector pCAMBIA2301, which contained the CaMV *35S* promoter, to generate the recombinant vector designated pCAMBIA2301-*CpCAF1*. The recombinant vector was transformed into *Agrobacterium tumefaciens* strain GV3103 and the kanamycin-resistant lines were selected. Transformation of Arabidopsis with *A*. *tumefaciens* was performed using the floral-dip method [61]. The T_1_ transgenic lines were selected on MS medium supplemented with 100 mg L^−1^ kanamycin. The kanamycin-resistant seedlings were transplanted to soil and were grown to maturation. The T_4_ transgenic lines were selected using the same method. Genomic DNA was extracted from the leaves of putative transgenic Arabidopsis plants using the Plant Genomic DNA Kit (TIANGEN) in accordance with the manufacturer’s instructions. The PCR amplification was performed with the primers *CpCAF1*-F/R. The PCR procedure included an initial preheating step at 95 °C for 5 min, followed by 27 denaturation cycles run of 95 °C for 30 s, annealing at 56 °C for 30 s, and an extension at 72 °C for 1 min 40 s, followed by a final extension time at 72 °C for 10 min. The ensuing PCR products were detected with 1% gel electrophoresis to confirm *CpCAF1*’s insertion into the transgenic plants. Real-time quantitative PCR was used to verify the identity of the transgenic plants and classify the transgenic lines into different groups based on the *CpCAF1* expression level.

### 4.6. Phenotypic Observation

To investigate whether *CpCAF1* affects the nutritional and reproductive growth of transgenic plants, we observed and recorded data on phenotypic traits at different growth stages of transgenic Arabidopsis and compared their growth rate and growth status with those of wild-type Arabidopsis. The T4 generation Arabidopsis strains identified as positive for the *CpCAF1* gene were subjected to expression analysis, and two transgenic strains (1 strongly and 1 slightly weakly expressed) were selected, and wild-type Arabidopsis (WT) was used as a control. In total, 90 plants of each transgenic strain and WT were selected as phenotypic observation record plants, and the phenotypes were observed and compared from seedling stage to scape-bearing stage to the decaying stage at regular intervals every day. In addition, the transgenic plants and WT were observed and photographed after 12 h of low-temperature (−4 °C) treatment and 12 h of resumption of normal culture (22 °C). Survival rates were then calculated after 3 days of recovery.

### 4.7. Measurement of Physiological Indicators

To assess the effect of *CpCAF1* on the physiological indicators of low-temperature treatment, rosette leaves were sampled from the transgenic and wild-type plants (each sample comprised 0.1 g). The proline content was determined following the method of Bates et al. [62]. The malondialdehyde (MDA) content was estimated using the procedure described by Heath and Packer [27]. Superoxide dismutase (SOD) activity was assayed by measuring the ability to inhibit the photochemical reduction of nitroblue tetrazolium in accordance with the method of Beauchamp and Fridovich [19]. Each measurement was replicated three times.

For ion flux analyses, transgenic and wild-type Arabidopsis seedlings germinated over 2 weeks were measured using the non-invasive microtest system (NMT-YG-100, Younger, Amherst, MA, USA). The ion-selective electrodes were calibrated using the following solutions: Na^+^, 0.5 and 5.0 mmol L^−1^ NaCl; K^+^, 0.05 and 0.5 mmol L^−1^ KCl [21]. Taproot segments were immobilized on the bottom of a measuring dish and incubated in the measuring solution to equilibrate for 5 min. Steady fluxes of K^+^ in the meristematic zone of intact roots were measured and continuously recorded for 10 min.

### 4.8. Cloning and Analysis of the Upstream Sequence of CpCAF1

Genomic DNA was extracted from the leaves of young seedlings using the cetyltrimethylammonium bromide (CTAB) method [58]. Using the *Chimonanthus salicifolius* genome [31] assembly as the reference, the upstream sequence of *CpCAF1* was amplified using the gene-specific primers *CpCAF1*-Pro-F (5′-GTGTAAGTGAAGAATTAAGAATTGTCG-3′) and *CpCAF1*-Pro-R (5′-CTTCTTCTCTTTTCCTTTTTTTTCCT-3′). The amplified fragment was designated *CpCAF1pro*. The purified product was cloned into the pMD19-T vector (TaKaRa Bio, Dalian, China), and then sequenced and analyzed. The prediction of *cis*-acting regulatory elements in the upstream sequence of *CpCAF1* was performed using the PLACE (http://www.dna.affrc.go.jp/PLACE/signalscan.html (accessed on 10 January 2021) and PlantCARE (http://bioinformatics.psb.Ugent.be/webtools/plantcare.html (accessed on 10 January 2021) databases.

### 4.9. Construction and Transformation of CpCAF1 Promoter Deletions Fused to GUS

For analysis of the *CpCAF1* promoter in transgenic tobacco plants, constructs containing the *CpCAF1pro::GUS* cassette were used. A 2133-bp promoter fragment (−1908 to +224 from the transcription start site; TSS), was amplified using specific primers (Table S2) containing the *Xba*I and *Nco*I restriction sites and cloned into the pCAMBIA1305.1 vector. Three truncated promoter fragments were generated and inserted into the vector using the same method: *CpCAF1pro*-P1 (−1980 to +2), *CpCAF1pro*-P2 (−1646 to +224), and *CpCAF1pro-*P3 (−941 to +224). The plasmids (*CpCAF1*pro/-P1/P2/P3::GUS) were transformed into Arabidopsis (Col-0) using the floral-dip method [19] mediated with *Agrobacterium tumefaciens* strain GV3101 cells. The seeds from T_0_ transgenic plants were selected on MS medium supplemented with 25 mg L^−1^ hygromycin B. Transgenic Arabidopsis T_2_ seeds were sown and the seedlings were cultured in MS liquid medium. Sampling was conducted after incubation for 6 h with three replicates per sample.

### 4.10. Histochemical and Fluorometric Analysis of GUS Activity

For histochemical and fluorometric assays of β-glucuronidase (GUS) activity, fresh samples (small plants or tubers) were treated with X-Gluc solution (Sigma-Aldrich, Shanghai, China). The GUS activity was quantified using 4-methylumbelliferyll-β-D-glucuronide (Sigma-Aldrich) as described previously [16]. The total protein activity was determined using Varioskan Flash (a full wavelength scanning multi-function reading device) in accordance with the method of Bradford [26].

### 4.11. Statistical Analyses

All statistical analyses were conducted with GraphPad Prism version 9.0.0 (GraphPad Software, Inc., San Diego, CA, USA).

## 5. Conclusions

In conclusion, the present findings showed that wintersweet *CpCAF1* accelerates each growth stage in Arabidopsis and specifically implied that the gene plays a role in promoting flowering through gibberellin or the autonomous pathway. Furthermore, *CpCAF1* enhances the low-temperature tolerance of *Chimonanthus praecox* by increasing the proline content, decreasing the MDA content, enhancing SOD activity, and increasing potassium ion flux, which supports the hypothesis that *CpCAF1* is a cold-related transcription factor with a positive regulatory effect in low-temperature tolerance enhancement. However, its specific regulatory mechanism still needs further study. In addition, the *CpCAF1* promoter is predicted to be a constitutive promoter containing elements involved in low-temperature stress response, which further supports the above conclusions. Given that *CpCAF1* is indicated to play important roles in flower development and low-temperature tolerance, the next logical step is to investigate the regulatory mechanism of the gene in promoting plant growth and earlier flowering and flower senescence, as well as the structural function of proteins encoded by *CpCAF1* and the potential regulators of *CpCAF1* in low-temperature tolerance.

## Figures and Tables

**Table 1:** Phenotypic traits in two T_4_ transgenic lines of *Arabidopsis thaliana* overexpressing *CpCAF1* and the wild type.

Phenotypic Trait	Wild Type	L5	L3
First rosette leaf collateral branch appeared (d)	36.33 ± 0.58 ^a^	33.00 ± 1.00 ^c^	34.33 ± 0.53 ^b^
First lateral branch of the stem appeared (d)	32.67 ± 1.53 ^ab^	28.33 ± 1.53 ^c^	31.00 ± 1.00 ^b^
First inflorescence appeared (d)	29.00 ± 1.00 ^a^	26.00 ± 0.07 ^d^	27.00 ± 1.73 ^bc^
First flower opened (d)	33.67 ± 1.53 ^a^	28.33 ± 0.58 ^d^	30.67 ± 0.58 ^b^
First silique appeared (d)	36.33 ± 1.15 ^a^	30.67 ± 0.58 ^c^	33.00 ± 1.73 ^b^
First yellow leaf appeared (d)	39.33 ± 0.58 ^a^	36.33 ± 1.53 ^cd^	37.00 ± 1.00 ^bc^

Each value is the mean ± SD calculated from at least three biological replicates. The significance of differences between the treatment and the control group was assessed with one-way ANOVA. Different lowercase letters within a column indicate a significant difference (*p* < 0.05).

**Table 2:** *Cis*-acting regulatory elements predicted in the promoter region of *CpCAF1*.

No.	Regulatory Element	Site	Sequence	Function of Site
1	ABRE	−986, −374	TACGTG	*Cis*-acting elements involved in abscisic acid response
2	ARE	−1741	AAACCA	*Cis*-acting regulatory element essential for anaerobic induction
3	AT1-motif	−538	ATTAATTTTACA	Part of a light-responsive module
4	AuxRR-core	−1329	GGTCCAT	*Cis*-acting regulatory element involved in auxin responsiveness
5	Box 4	−667, −491	ATTAAT	Part of a conserved DNA module involved in light responsiveness
6	W-Box	+124, −368	TTGACC	WRKY transcription factor binding site involved in defense responses
7	DRE	−456	TTCGACC	Induced by both drought and low temperature
9	ERE	−1623, −1058	ATTTCAAA	Ethylene-responsive element
10	G-Box	−987, −374	CACGTT	*Cis*-acting regulatory element involved in light responsiveness
11	GT1-motif	−1628	GGAGATG	Light-responsive element
12	L-box	−58	ATCCCACCTAC	Part of a light-responsive element
13	LTR	−249	CCGAAA	*Cis*-acting element involved in low-temperature responsiveness
14	MYB	+151, −186, −1231	TAACCA	Response to drought and salinity stress tolerance
15	MYC	−1107, −1159, −995	CATTTG	Response to drought and abscisic acid signals
16	SARE	−1050	TTCGACCATCTT	Salicylic-acid-responsive element
17	STRE	+143, −173, −207, −342	AGGGG	Stress-responsive elements
18	Sp1	−66	GGGCGG	Light-responsive element
19	TCA	−46	GAGAAGAATA	*Cis*-acting element involved in salicylic acid responsiveness
20	TCT-motif	−1464	TCTTAC	Part of a light-responsive element
21	WUN-motif	−1830	CCATTTCAA	Injury-related element
22	Telo-box	+172	AAACCCTAACCCTAA	Motifs associated with MYB binding sites

## Data Availability

The data presented in this study are available in the article.

## Supplementary Materials

The supporting information can be downloaded at: https://www.mdpi.com/article/10.3390/ijms241612945/s1.
