# Supplementary material for: CpCAF1 from Chimonanthus praecox Promotes Flowering and Low-Temperature Tolerance When Expressed in Arabidopsis thaliana
Source: Int J Mol Sci. 2023 Aug 18;24(16):12945. doi: 10.3390/ijms241612945 (PMC10455127; doi:10.3390/ijms241612945)
Supplement: Supplementary file 1 [file ijms-24-12945-s001.zip › ijms-2528667-supplementary.pdf]

Table S1. The primers designed for real-time quantitative PCR.

Primer name	Primer sequence (5′-3′)
qCpCAF1-F	CAAATCAGAGAGGTATGGAACGACAAT
qCpCAF1-R	GTAGTCGGACGTGGTCTTGAAGTTG
CpActin-F	GTTATGGTTGGGATGGGACAGAAAG
CpActin-R	GGGCTTCAGTAAGGAAACAGGA
CpTublin-F	TAGTGACAAGACAGTAGGTGGAGGT
CpTublin-R	GTAGGTTCCAGTCCTCACTTCATC
AtActin-F	GTTCGTCTTCCACTTCAG
AtActin-R	ATCATACCAGTCTCAACAC
AtFT-F	TTCCAAGTCCTAGCAACCCTCACC
AtFT-R	TTCTTCCTCCGCAGCCACTCTC
AtFLC-F	CCTAATTTGATCCTCAGGTTTGGG
AtFLC-R	CCGACGAAGAAAAAGTAGATAGGCAC
AtSOC1-F	GCTCTCAGTGCTTTGTGATGC
AtSOC1-R	A AGAACGTACTTGGAGCTGGC
AtELF3-F	CTGGACCATCTAGTCAGCCTTG
AtELF3-R	TGCCATGAGACTGAGATCTTCTTG

Table S2. PCR Primers used for cloning *CpCAF1* promoter and deletion fragment.

Name	Primer sequence (5′-3′)	Distance to TSS
CpCAF1pro-F	GC**TCTAGA**GTGTAAGTGAAGAATTAAGAATTG	−1908
CpCAF1pro-P2-F	GC**TCTAGA**GAAACTGATGTCAAATTGGGTTAAT	−1646
CpCAF1pro-P3-F	GC**TCTAGA**TAAGGTTCAATACTTCTGTGACATG	−941
CpCAF1pro-R	CATG**CCATGG**CTTCTTCTCTTTTCCTTTTTTT	+224
CpCAF1pro-P1-R	CATG**CCATGG**CGGGAGGGCTAGGGAAAG	+2

Note: The restriction sites are indicated in bold, *Xba*I: TCTAGA , *Nco*I: CCATGG.
